# Design of an RRAM-Based Joint Model for Embedded Cellular Smartphone Self-Charging Device

**DOI:** 10.3390/mi16101101

**Published:** 2025-09-28

**Authors:** Abhinav Vishwakarma, Anubhav Vishwakarma, Matej Komelj, Santosh Kumar Vishvakarma, Michael Hübner

**Affiliations:** 1 Computer Engineering, Brandenburgische Technische Universität, 03046 Cottbus, Germany; michael.huebner@b-tu.de; 2 Department K-7, Jožef Stefan Institute, 1000 Ljubljana, Slovenia; anubhav.vishwakarma@ijs.si (A.V.); matej.komelj@ijs.si (M.K.); 3 Department of Electrical Engineering, Indian Institute of Technology, Indore 453552, India; skvishvakarma@iiti.ac.in

**Keywords:** CMOS, energy harvesting, emerging applications, permanent magnets, Resistive RAMs (RRAMs), smartphones, self-charging, wireless-sensor

## Abstract

With the development of embedded electronic devices, energy consumption has become a significant design issue in modern systems-on-a-chip. Conventional SRAMs cannot maintain data after powering turned off, limiting their use in applications such as battery-powered smartphone devices that require non-volatility and no leakage current. RRAM devices are recently used extensively in applications such as self-charging wireless sensor networks and storage elements, owing to their intrinsic non-volatility and multi-bit capabilities, making them a potential candidate for mitigating the von Neumann bottleneck. We propose a new RRAM-based hybrid memristor model incorporated with a permanent magnet. The proposed design (1T2R) was simulated in Cadence Virtuoso with a 1.5 V power supply, and the finite-element approach was adopted to simulate magnetization. This model can retain the data after the power is off and provides fast power on/off transitions. It is possible to charge a smartphone battery without an external power source by utilizing a portable charger that uses magnetic induction to convert mechanical energy into electrical energy. In an embedded smartphone self-charging device this addresses eco-friendly concerns and lowers environmental effects. It would lead to the development of magnetic field-assisted embedded portable electronic devices and open the door to new types of energy harvesting for RRAM devices. Our proposed design and simulation results reveal that, under usual conditions, the magnet-based device provide a high voltage to charge a smartphone battery.

## 1. Introduction

Recently, many unique memory techniques have been investigated [1]. Integrated circuits’ ongoing reduction in cost and power consumption has been the primary driver of the explosive growth in portable electronic devices. The prospective flow of hybrid technology is predicted to be a leading-edge technology in the forthcoming years [2]. In this situation, resistive random-access memory (RRAM) is a possible contender for next-generation Non-Volatile Memory (NVM) technology [3]. This technology is defined by its accessible structure, quick switching speed, great scalability, and integration ability at a standard CMOS process’s Back-End-Of-Line (BEOL) [4,5]. As a cutting-edge electronic component, the memristor has enormous potential for energy-efficient technologies in the future, especially for embedded electronic portable systems [6]. The research for memristor devices with excellent energy efficiency is still ongoing, and a recently created self-generating technology that can capture different types of environmental energy to power functional components showed commitment [7]. An external-power-supply-source dependence is eliminated by this self-powered technology. This self-powered memristor gadget satisfies the consequence approach and application prerequisites of green electronics and has the benefits of being environmentally benign, sustainable, and renewable [8,9]. The literature survey indicates that integrating and combining several devices is a potential technique for multipurpose, power-free, smart electronics [10]. This new device element has gained a widespread adoption and a lot of popularity in recent years because it has the potential to integrate these self-powered systems with other functional devices and power-free electronics, like self-powered motors, sensors, detectors, generators, and energy-efficient memristive systems [11].

Over the past few years, numerous researcher have developed a variety of self-powered technologies that sense physical conditions, including moisture-powered memristive systems and various technologies [12]. The aforementioned power-free electronics systems served as inspiration for the incorporation of memristors, the fundamental sensor unit, into self-powered technologies [13]. In further research and development, memristor-based solutions may open the door to more environmentally friendly and self-sufficient mobile devices, revolutionizing smartphone powering and drastically lowering the demand for conventional charging techniques [14]. Therefore, wireless sensor nodes require alternate charging sources. The literature has proposed generators based on piezoelectric [15], electromagnetic [16], and electrostatic [17] conversion. Therefore, developing self-powered memristive devices would be highly relevant and remarkable.

This study presents a memristor model for an internal generation system for self-charging a smartphone’s battery. Self-powered embedded portable devices are architecturally constituted of an electromagnetic nanogenerator and a memristive division which are connected together in series [18]. This concept can serve as the building block for a conventional SRAM cell if a higher resolution is achieved with a self-powered memristive device to investigate the modeling and design of a magnetic induction caused by an oscillating permanent magnet mechanical-to-electricity converter to be used as a power source for the wireless self-charging devices. The reader is referred to the concept of the work by A. Vishwakarma et. al. [19,20,21] for a discussion of associated self-powered charging electronic devices. Although others have proposed the idea of using an RRAM device within an optimized CMOS memristor-based cell [22], these implementations are merely proposed as a concept, using an existing and specific RRAM device [22,23]. Our research is focused on the simulation application of the RRAM model device. By combining it with a magnetic field, we aim to generate enough output voltage for edge devices, making it an ideal fit for smartphone self-charging applications. A self-generating memristive system such as this may open up a whole new field of study that could significantly advance the creation of new technology.

**Figure 1:**
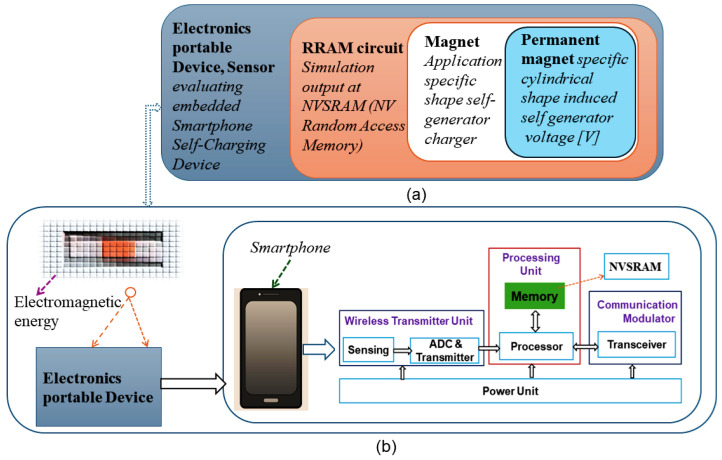
(**a**) Proposed concept: A circuit-level integration approach for RRAM-compute blocks for self-energy generation. (**b**) SoC Block diagram of various modules of the wireless smartphone self-charging device.

As a proof of concept we simulate the operation of RRAM units, included in a portable, smartphone-like device, schematically presented Figure 1a: A circuit-level integration approach for RRAM-compute blocks for self-energy generation Figure 1b SoC Block diagram of various modules of the wireless smartphone self-charging device. The simulation of the RRAM device uses a 1T2R cell design with an optimized cylinder-shaped permanent magnet; the proposed concept shows the auto energy generation for wireless smartphone self-charging applications. Hence, an improvement in total power is obtained. In this study, we present an implementation that consists of a memristor-based 1T2R optimized using IHP 130 nm SG13S technology and prove the concept by means of the finite-element magneto-static simulations that allow for the combination of RRAM device technology into CMOS designs.

This paper presents the following essential features:

- The proposed IT2R RRAM cell operates better than the previous design [22,23] in terms of write/read time and power consumption, and combining RRAM with IT2R cells is more efficient than using conventional memories.
- The suggested concept [19,20] is to generate self-energy by magnetic induction from a permanent magnet oscillating inside a coil.
- The successful simulation of the joint RRAM device and the proposed in-built permanent magnet validates its potential for smartphone self-charging. applications.

The rest of the paper is structured as follows: We introduce relevant topics, background, and related work in Section 2. Section 3 presents the methodology used for implementation. We evaluate the simulation and discuss the results in Section 4 and conclude the paper in Section 5.

## 2. Background and Related Work

### 2.1. Memristor Modelling

**Figure 2:**
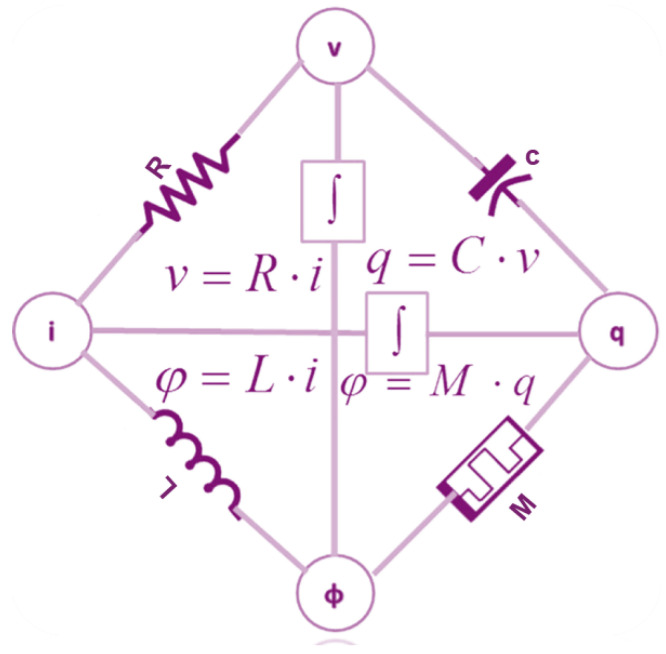
Combinations relationships among circuit with Memristance M [24,25].

**Figure 3:**
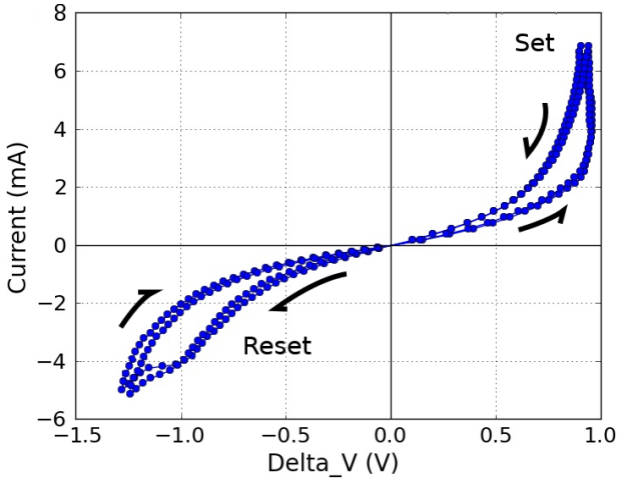
The I–V cycle hysteresis loop of a memristor [25,26].

**Figure 4:**
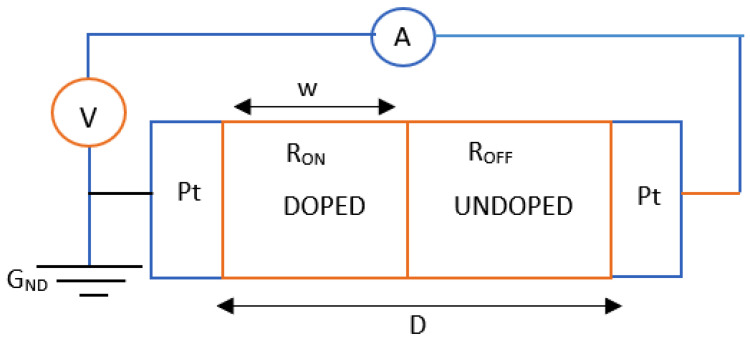
The memristor model based on linear ion drift titanium dioxide from HP Labs [24,29].

The memristor was introduced as a late-discovered detachable circuit component. In 1971, professor Leon Chua introduced the memristor, serving as the missing relationships between the six potential arrangements of the interactions of four essential circuit variables such as the voltage (v), current (i), flux (ϕ), inductor (L), R (resistor), capacitor (C), and electric charge (q) [24,25]. These possible combinations of the relationships are illustrated in Figure 2 [26]. In 2008, HP labs developed the utmost elementary titanium dioxide memristor; it is fundamentally in the light of a two-layer paltry “layers that are stacked” of titanium dioxide thin film ($TiO_{2}$), surrounded by dual platinum (Pt) electrodes. Titanium dioxide ($TiO_{2}$) is commonly employed in oxygen sensors after treatment with oxygen atoms due to its changing resistance [22]. The motion of electrons in that substance regulates the haphazard motion of these atoms in the tiny film, allowing for a change of state in the device’s nuclear framework or primarily in the memristor. The base layer serves as an insulator, while the topmost layer conducts due to extra oxygen vacancy in the ($TiO_{2}$) material [23,24]. This resistance or state changes when vacancies in oxygen move into the bottom layer; as a result, the topmost layer remains stable. Figure 3 illustrates the characteristic hysteresis curve of a memristor. A hysteresis curve may be produced by applying a DC signal, which includes the Vset and Vreset thresholds of the device. Z. Biolek, D. Biolek, and V. Biolkova [27] have proposed an early device model that is mathematically similar to [28]. The model was rather simple, allowing only one state variable. It was constructed as follows: The oxygen atomic doping approach in ($TiO_{2}$) skinny films generates two zones with varying resistances in series with the film [27,28]. While the nondoped region has higher resistance and poorer conductivity, the doped zone ($TiO_{2}$-X) has superior conductivity and lower resistance. This is illustrated by an element consisting of two resistors arranged in series ($R_{ON}$, $R_{OFF}$, where $R_{ON}$ < $R_{OFF}$). The state variable *w* denotes the proportion of each resistor’s doped or undoped portion (see Figure 4). Doped regions function as $R_{ON}$ and have an oxygen deficit ($TiO_{2}$-X), while undoped regions function as $R_{OFF}$ [23,24].

### 2.2. Related Work

Over the ensuing years, many sophisticated models have been invented. Xu Zhang et al. have developed a method for strategically planning mobile charging services for EVs that are in high demand. This study concentrated on scheduling mobile chargers rather than the current systems’ exclusive focus on service discipline for EV options [30]. Wu’s team developed a sophisticated neuromorphic tactile sensor based on a triboelectric nanogenerator. The device can simulate self-powered neuroplasticity without neuromorphic circuitry by incorporating a decreased graphene-oxide layer into the friction layer [31]. Olivier Djakou Nekui et al. examine the argument for electromechanical dampening; the quadratic n is based on an IoT-based pulse rate-monitoring gadget that the author constructed to remotely monitor patients with unstable medical conditions [32]. A. Vishwakarma and his colleagues explored the applicability of a magnetic-induction-based charger, made of permanent magnets, for charging smartphone batteries. In the context of the finite-element method, this investment is concentrated on vibrating interest and computing the induced voltage [19]. C. R. Saha et al. developed this approach to describe an electromagnetic-based generator that may produce power from human body motion and provide energy for body-worn sensors or electronic equipment. The generator model was constructed and examined using a shaker under timbre conditions and human body movement when striding and jogging leisurely [33]. Semyung Park et. al. focused on internal-type little linear generators, and this inversion demonstrated that the vibrational model is investigated using mechanical resonance, and the magnetic circuit, consisting of a permanent magnet, steel yoke, and coil, is constructed to boost electricity generation [34]. Liu et al. described a self-generating artificial sense memory device that combined a triboelectric nanogenerator (TENG) and a field effect synaptic transistor (FEST) [35]. Ghulam Dastgeer et al. demonstrated that our SnSe2 memory devices can store multi-bit data. Recent advancements in non-volatile memory devices have highlighted their promise for high-capacity and reliable data storage [36]. Ghulam Dastgeer et al. revealed that this study has thoroughly explored advances in perovskite solar cells (PSCs), with an emphasis on material properties, configurations, fabrication procedures, and emerging trends [37]. Abhinav et al. designed and investigated RRAM devices that can be utilized as (re)programmable comparators, resulting in a non-uniform sampling ADC for sensor data [29]. Jiang et al. [38] presented the Stanford PKU model, which Reuben et al. [39] adapted to reflect RRAM devices fabricated at IHP. Subsequently, we decided to use it for our research.

### 2.3. Existing Structures of NVSRAM Cells

**Figure 5:**
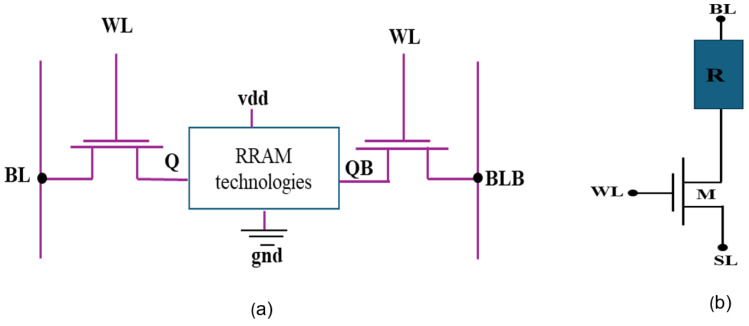
(**a**) Structure for the unique hybrid construction of NVM with a 6T SRAM cell core. (**b**) Existing 1T1R [1,5].

**Figure 6:**
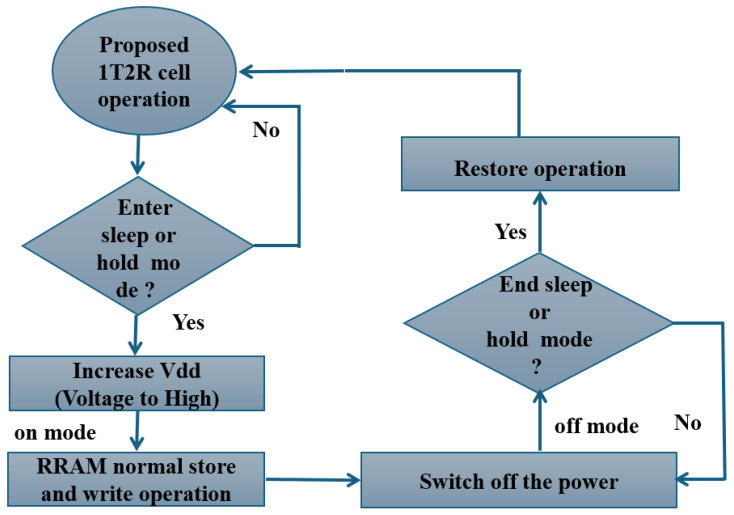
The operational flow chart of an 1T2R RRAM cell [41].

Figure 5a illustrates the structure model for the unique hybrid construction of NVM with an RRAM built into a 6T SRAM cell core. Non-volatile SRAM (NVSRAM) was created by combining SRAM and RRAM technologies. The SRAM cell’s data terminals are linked to the RRAM resistive element, which allows data backup functions while maintaining SRAM standard mode functionality (i.e., hold, write, and read operations) [1]. Storage and restore operations are carried out before turning off the power and after turning it back on, respectively [40]. Figure 6 depicts the operational working flow chart for the presented 1T2R cell. The process of creating a working flow NVSRAM has been explained by others [41]. In standard mode, the NVSRAM cell behaves similarly to an SRAM cell, including read and write operations [1,41]. The existing designs of non-volatile SRAM cells are illustrated in Figure 5.

#### 1T1R

The 1T1R-based NVSRAM cell [5] is shown in Figure 5b. The 1-transistor–1 resistor (1T1R) configuration has been utilized extensively in this application, with the OxRAM cell connected on top of the transistor, as illustrated in the diagram. The insulator layer, also known as the switching layer, is one of the three layers of the OxRAM cell that work as storage. It is positioned between two metallic electrodes, the top electrode TE and the bottom electrode BE [5]. The CMOS transistor is a popular picker used with RRAM technologies. Furthermore, the semiconductor transistor protects memory cells from harm during programming operations by limiting the acceptable current passing within the OxRAM cell.

### 2.4. Energy Generation System

#### The Self-Generating System Based on Electromagnetic Mechanisms

Based upon Faraday’s Law, oscillating magnetic fluxes generate an electric field in a winding, corresponding to the fundamental concept of electromagnetic induction. The magnetic-field intensity, velocity, affect the quantity of electricity generated [19]. Faraday’s law defines that the induced voltage is expressed as a derivative of time (t) and magnetic flux (ϕ).

Faraday’s law defines the time (t)-dependent induced voltage V(t) [19,20]:

(1) $$\begin{array}{c} v\left(t\right)=-N\frac{d\phi }{dt} \end{array}$$

(2) $$\begin{array}{c} d\phi =NBA \end{array}$$

where N is the number of turns in the coil, A represents the coil’s cross-section, and B indicates the magnetic flux density.

**Figure 7:**
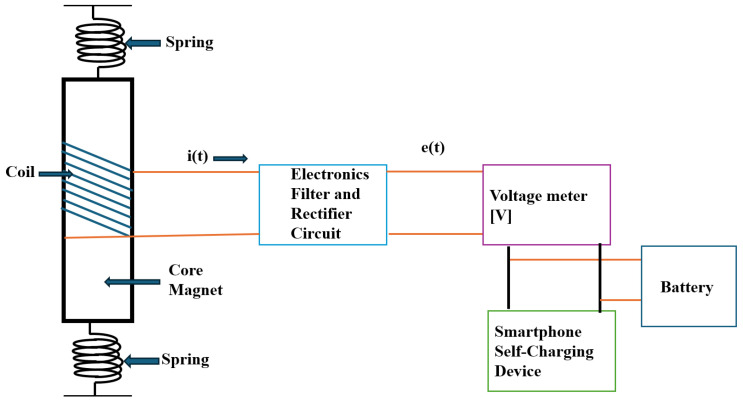
A principle scheme of an induction-based charger [19,20].

Figure 7 depicts a diagram of the process of energy conversion principle. The considered self charger consists of a rectifier circuit and a coil surrounding a magnet attached to a pair of strings. The electromotive force is produced at the terminals of the stator coils when the slider moves up and down due to the heaving action of the smartphone, which is brought on by mechanical energy from the user’s walking and shaking motion [20]. While the springs must be optimized to match the walking and oscillating magnet frequencies, and the rectified electric circuit must be designed to transform the voltage signal properly, this paper aims to find the best shape of the magnet that generates an oscillating magnetic field.

The battery output is fed into the self-charging circuit device, which consists of three main components: a rectifier circuit used for rectification to convert AC to DC conversion, voltage meter amplification used for high gain output voltage, and a smartphone battery. When an external vibration is stimulated, a magnet on a single-plane spring oscillates near the stationary coils, altering the magnetic flux inside the coils and producing voltage. The electronic filter used by the transducer converts the vibrational energy into electrical energy. The output is now fed into the rectifier circuit to convert it into DC signals in pure, continuous form. The achieved output after regulation is adequate to charge a smartphone and power tiny electronic gadgets. The circuit simulation arrangement is in the following Sections.

## 3. Methodology

In this section, we explain how we constructed an optimized 1T2R (one-transistor-two-resistor) and extended the circuit toward a permanent magnet transition into a device with self-energy generation. This approach demonstrates significant progress in developing efficient, self-charging embedded electronic devices.

### 3.1. Concept

This section is concisely and precisely describes the Conventional 6T SRAM cell and proposed design structure.

#### 3.1.1. The Conventional 6T Cell

**Figure 8:**
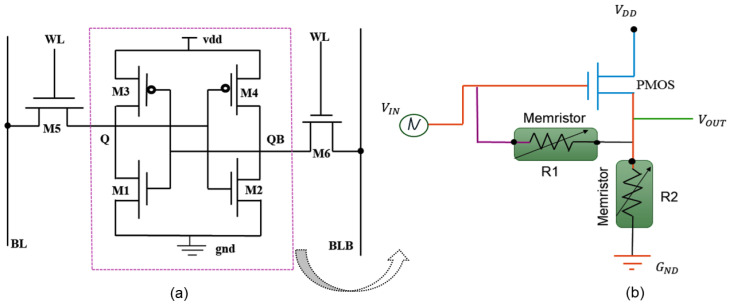
(**a**) Conventional 6T SRAM cell structure [10] (**b**) Proposed 1T2R RRAM-based structure.

A single SRAM memory cell consists of six transistors (M1, M2, M3, M4, M5, and M6). A standard SRAM cell is formed by a latch (using two cross-coupled inverters) and two access transistors. Figure 8a depicts a schematic view of conventional 6T SRAM, with the cross-coupled inverters M1–M3 and M2–M4 [10]. The two access transistors, M5 and M6, are coupled to the complementary bit lines BL and BLB. The primary operations of an SRAM memory cell are hold, read, and write, which are carried out using Word Lines (WLs) and Bit Lines(BLs). Every SRAM cell has the potential to store “0” or “1”. WLs are connected to the gates of the selected transistors (M5 and M4), while BLs are attached to the transistor terminals. During the hold stage, the WLs are deactivated, disconnecting the BLs from the SRAM core [1]. Data is stored in the latch structure on data nodes Q and QB [5]. WL is activated to perceive or update the stored data in order to complete read and write activities. An SRAM has three major operations: hold or standby, read, and write mode.

- Hold or Standby mode: During standby mode, the world line signal deactivates the access transistors, disconnecting the bit lines voltage from the storage nodes [42].
- Read Mode: During read operation, the WL signal remains active to switch on the access transistors, and the bit lines are pre-charged to vdd. The storage node Q supplies a discharge channel to the matching bit line BL, and the sense amplifier at the read output port detects the voltage difference between the two bitlines [10].
- Write Mode: The bit line is used with the value of be stored in SRAM. The world line control signal activates the cell through the access transistors, which can change the last state of the cross-coupled inverter with the weaker transistor. Therefore, the substitute value is saved [42].

#### 3.1.2. Proposed 1T2R Structure

In this section, we introduce the proposed schematic of the 1T2R design in cadence virtuoso 130 nm software, as illustrated in Figure 8b. This architecture has been invented using twin memristors (R1 and R2) and a transistor. It is a volatile memory device with cross-coupled inverters (seen in Figure 8a), which are replaced by the RRAM-based design and the storage units of this circuit, which act as two access transistors. The proposed circuit employs a single P-channel metal oxide semiconductor (PMOS) and dual memristors (R1 and R2) instead of an NMOS with a lower threshold voltage to increase switching speed. The design comprises a series of PMOS transistors associated with an insulator-metal. Memory units regulate the current that flows via PMOS and are utilized for recent feedback. The provided voltage $V_{DD}$ is connected to the PMOS transistor’s source terminal, as indicated in the schematic—these mechanism designs, including memristor R1, serve as a feedback branch. This circuit are simulated using the usage window function in conjunction with the linear boundary drift model. We utilized the Stanford PKU memristor model parameters as described in [39,43] and illustrated in Table 1. The details of the conventional 6T SRAM design part have been clarified in the earlier section. Conventional SRAM can perform only read, write, store, and write/write activities, While NVSRAM can read, write, store, and recall. Table 2 shows the transistors’ dimensions and the parameters fed to the memristor model to simulate the behavior of the IHP’s 130 nm technology RRAM cells. While others have already suggested this idea and the subsequent equations [22,23], we contribute by extending this idea to a particular CMOS process and a specific RRAM device. Afterward, the second memristor (R2) is applied to connect the output node to the ground. In the previously mentioned states of our circuit, this memristor is positioned to regulate the voltage division and the PMOS transistor’s passing current. The positive terminals of the memristors, R1 and R2, are connected to the output node. This circuit uses a PMOS as a switching component. Because $V_{DD}$ is constant, the PMOS switch’s on and off states are scheduled using the input voltage $V_{IN}$. The current will flow through the memristor with activation of $R_{ON}$ if the input voltage $V_{IN}$ is adjusted to zero, placing PMOS, R1, and R2 in an on state (shown in Table 2). If the source-to-gate voltage falls lower than the PMOS threshold voltage, memristor R1 switches off while R2 remains on, indicating a PMOS cutoff. When $V_{IN}$ = 0, the transistor enters the on-state, extracting Equation (4), indicating the output voltage described below [22,23], where Req is the corresponding memristance of the memristor.

(3) $$\begin{array}{c} V_{0UT}=R_{eq}\cdot I_{D} \end{array}$$

(4) $$\begin{array}{c} V_{0UT}=\left(\frac{R2}{R2+R1}\right)\cdot V_{1}=\left(\frac{R_{ON}}{R_{ON}+R_{OFF}}\right)\cdot V_{1} \end{array}$$

Consequently, it is possible to ascertain the PMOS transistor’s passing current and output node voltage. According to our memristor model, the $R_{ON}$ and $R_{OFF}$ values are different. Both memristors stay in the on state because the current flows to their positive sides. Memristors with identical resistance and current could be used to extract an output power, which would be 1.5 V at that level. If 1.5 V is the input voltage, the condition will change. If the voltage difference (VSG) is less than the edge voltage (VTP) of the PMOS transistor, the PMOS switch is off. While memristor R2 stays in the on position, memristor R1 stays in the off position due to the electrical current produced by the input voltage. Acquiescent resistance $R_{OFF}$ generates a circumstance with no current flow. In (3), the voltage output is as follows [22,23]. By changing the actual values of $R_{ON}$ and $R_{OFF}$, it is clear that the result node is set to roughly 0 volts. In other words, such a design approach aims to achieve the same goal as the perfect ideal non-volatile output. The simulation results are presented in the next section.

#### 3.1.3. Proposed Novel Schematic Design for the Self-Energy Generator

**Figure 9:**
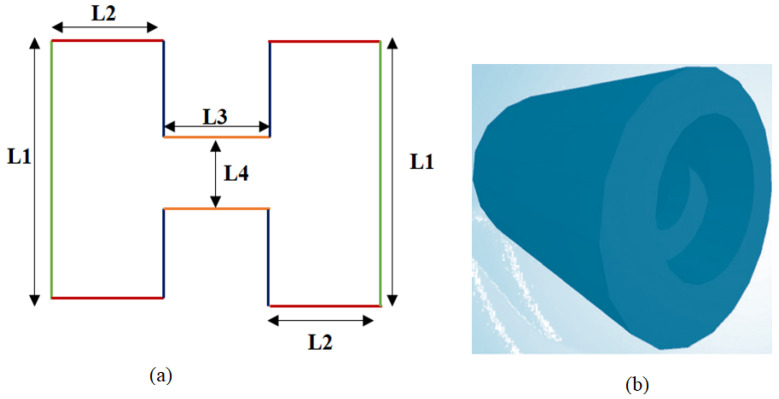
(**a**) The schematic and dimensions represent the proposed permanent magnet generator. (**b**) The proposed shape of the magnet.

The main idea is to use a single magnet of a specially-designed shape, magnetized uniformly in one direction. In Figure 9a, we illustrate the shape and structure of our proposed permanent magnet. The cylindrical shape with notches (Figure 9b) under consideration allows for the adherence to a planar problem because of its rotational symmetry. Table 3 displays the geometric details.

**Figure 10:**
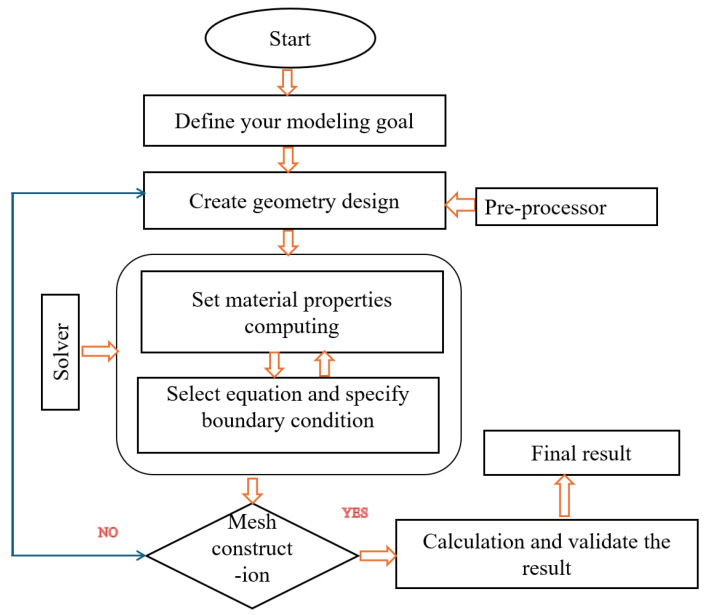
Detailed flow procedures for the magnet-modeling.

**Figure 11:**
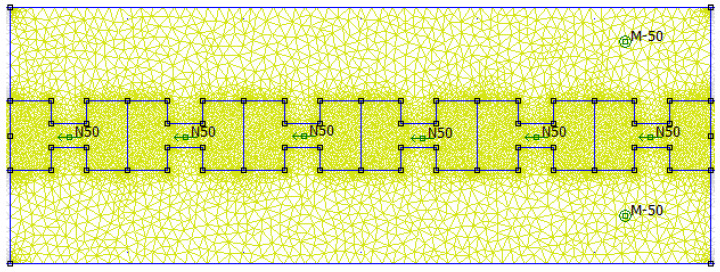
The software design schematic represents the proposed permanent magnet self-energy generator, with green arrows indicating magnetic direction.

Figure 10 depicts the entire procedure diagram for the magnetization representing technique. We adopt the magnetic characteristics of a cutting-edge sintered magnet (NdFeB-50) with a high enough remanent magnetization. The specially designed notches in the magnet reduce the weight and improve the required inhomogeneity of the resultant field, even for uniaxial magnetization, as shown in Figure 11, which is essential for a non-zero time derivative in Equation (1).

The FEMM software (https://www.femm.info/wiki/HomePage) was used to perform FEM computations of magnetic-flux density. The mesh design and application of the boundary constraints must require special consideration. A triangular mesh and the first-type (Dirichlet) boundary conditions were used. The convergence tests were used to calculate its density [20]. The meshing for the magnet geometry under consideration is shown in Figure 11. The mesh structure is tighter toward the magnet’s surface, where field gradients are more pronounced. The time dependence of the calculated flux is modeled by examining various displacements between the coil and magnet while assuming harmonic motion [19]. To simplify the process, the angular frequency was fixed to $\omega =1s^{-1}$. Equation (1) is carried out in terms of a finite-difference method to the time derivative, and Equation (2) applies the average value B of the magnetic flux density for a given cross-section. The main advantage of these suggested magnets is that they produce a sufficient voltage for charging an electronic device battery in normal circumstances. The evaluations are presented in the next section.

## 4. Simulation Results and Discussions

### 4.1. 1T2R RRAM Simulation

**Figure 12:**
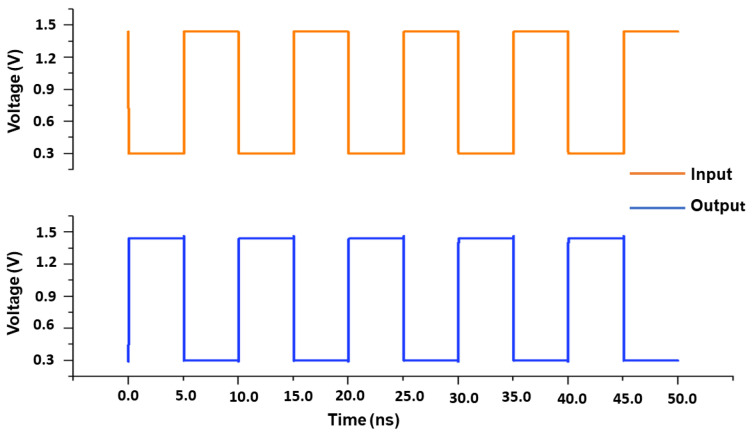
Transient analysis of the proposed design.

**Figure 13:**
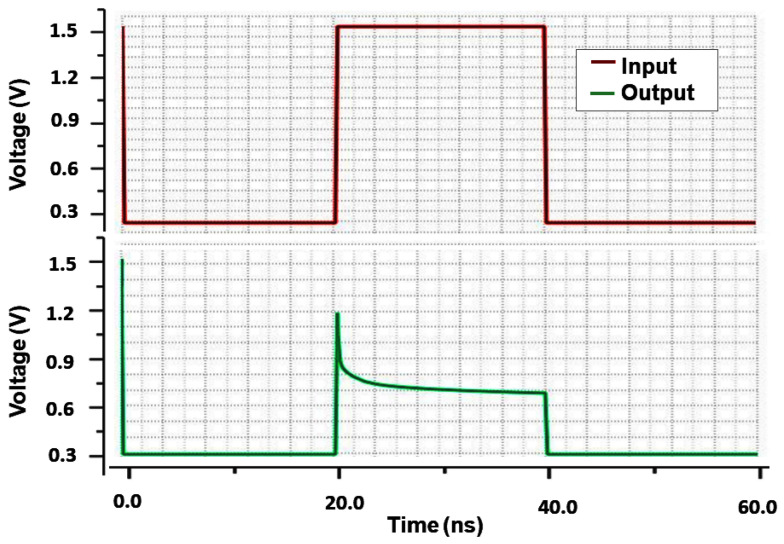
Proposed design non-volatile output write time analysis.

**Figure 14:**
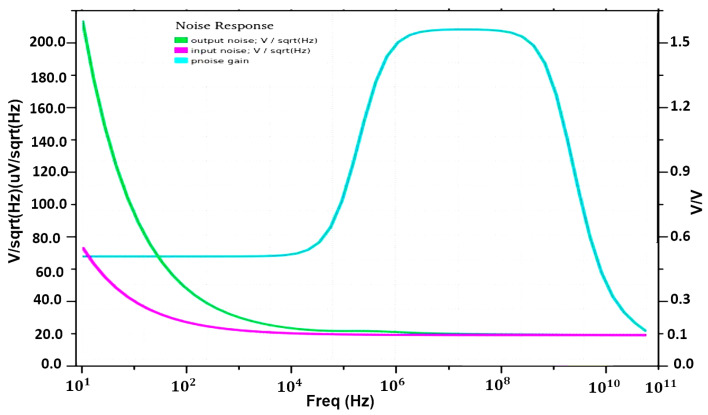
Noise response of our design.

This part demonstrates the usefulness of optimizing our proposed structure for usage with RRAM electronic devices. As previously explained, the suggested 1T2R architecture can keep its pre-programmed state, operating similarly to traditional SRAM cell behavior. The designed 1T2R and other reference circuits have been modified and simulated using industry-standard 130 nm CMOS technology and device size. The simulation results given were obtained using Cadence Virtuoso. The entire simulations are performed considering 1.5 V of the input voltage at the operational temperature of T = 27 °C while consuming a total power of 17.67 µW. A memristor-based feedback system has been used to analyze the read/write operation and non-volatility output. The circuit has a non-volatile output that is memristor-received. To examine the non-volatility characteristics of the proposed design, the most critical concerns, such as read and write time concerns, should be kept in mind. The output memristor (R2) is connected to the input memristor (R1) through a feedback-wired loop network. As in the state of R1, the input values are defined. The voltage at the output node is tuned to around 0 volts whenever the actual values of $R_{ON}$ and $R_{OFF}$ are substituted. This indicates that the circuit functions similarly to an ideal inverter. The transient analysis of the presented design is displayed in Figure 12. The rise and fall times are 5 ns. The various memristor variables for $V_{IN}$ = 1 and $V_{IN}$ = 0 and the proposed design based on memristor are shown in Table 2. The writing term is the shortest possible duration of time the input pulse must have been present for the memristor state to change from w = 0 ($R_{OFF}$) to = D ($R_{ON}$) [23]. In the suggested circuit, the R2 memristor will stay at $R_{ON}$ after the write time has elapsed. Following the writing time, if the input voltage $V_{IN}$ causes the output response for $V_{OUT}$ = 1, the memristor R1 will change. In this particular scenario, all memristors are enabled. Whenever $V_{OUT}$ = 0, the memristor condition is fixed allowing the significances in Table 2. Under these circumstances, R2 is on and R1 is off. In this case, the zero output value is expected. Figure 13 displays the simulation outcome for this condition. It will be beneficial in defining the circuit’s routine as well as the memristor R1’s intended function as an SRAM cell. Circuits develop non-volatile performances as a result. Figure 14 presents the noise performance simulation results, illustrating the impact of input-referred noise, output noise, and phase noise margin for the suggested design. The correlation between frequency (Hz) and V/sqrt referred noise (V/Hz) is displayed in the graph plot. Table 4 shows the measured parameters of the presented RRAM design. The suggested system design is an innovative concept that focuses on efficient memory utilization for self-charging devices.

#### Monte Carlo and Voltage Transfer Characteristic Simulation

**Figure 15:**
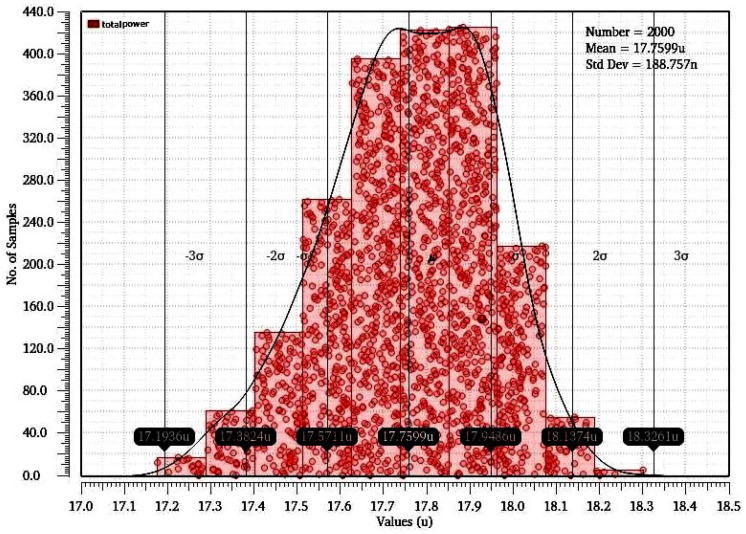
Power consumption results from Monte Carlo simulations.

**Figure 16:**
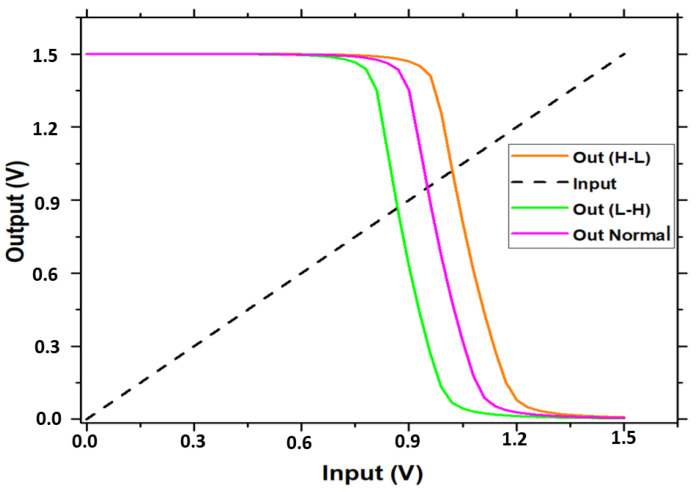
Voltage Transfer Characteristic of our design.

Monte Carlo (MC) is a popular statistical technique for calculating the probability of certain events under an unknown distribution. Figure 15 shows the results of Monte Carlo simulations of 2000 samples for the total power utilization of the suggested RRAM memristor-based design under different processes and mismatch operations. The overall power usage is around 17.75 µW, with a minor standard deviation of 1.85 µ. We evaluate the hysteresis curve for the proposed circuit, as depicted in Figure 16, and point to the outputs H-L and L-H. This curve demonstrates enhanced performance when bootstrapping the voltage drop. These circuits under consideration have a high-to-low voltage transfer characteristic (VTC) transition. H-L and L-H are the circuit’s upper and lower threshold voltages; outnormal is the input and output crosspoint voltage. Table 5 presents a comparison between the suggested RRAM (1T2R) design and designs that have been proposed in the literature.

### 4.2. Magnet Self Generator Optimizations

**Figure 17:**
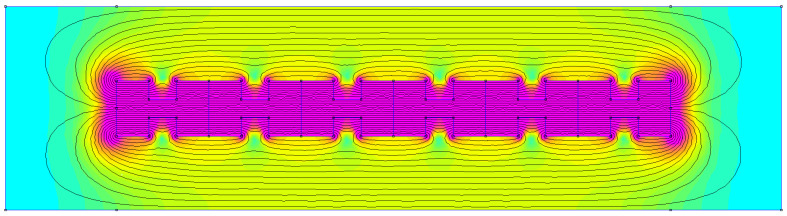
The calculated flux lines permanent shape magnet in the equilibrium position.

**Figure 18:**
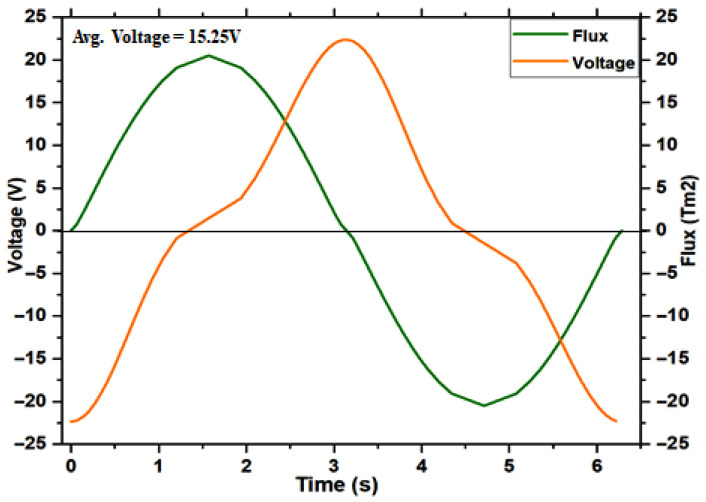
The computed difference between the permanent magnetic flux at equilibrium and the induced voltage.

**Figure 19:**
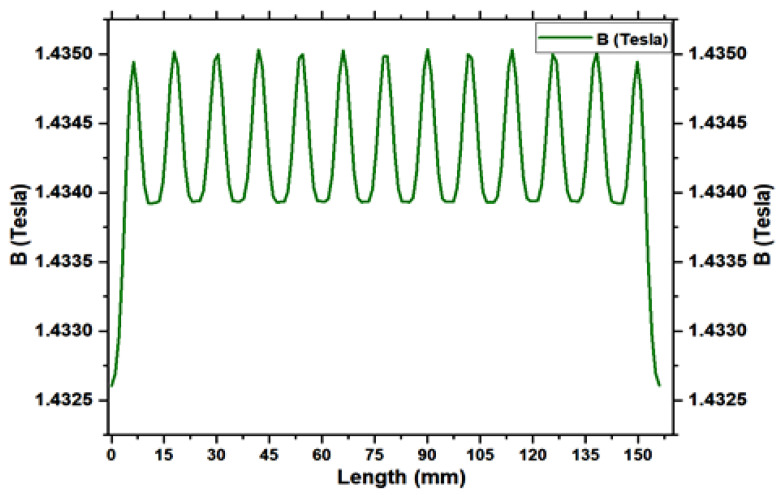
The calculated flux density in Tesla.

In this section, we will evaluate the self-generation system through numerical simulation with finite-element methods; to prove that the proposed solution may be successfully implemented in a self-charging device. We use FEMM Ansys software to achieve the simulation results that are depicted. The application of the permanent magnet for self-energy generation part has been explained in the previous section. Based on this assumption led to the use of the law of motion from the Maxwell equation for the overall investigation. It can charge the battery in both walking mode and shaking mode. Walking motion generates mechanical energy that may be transformed into electric energy, continually charging the battery and prolonging its useful life. The permanent magnetization is constant and aligned with the horizontal axis, which equals the oscillation direction. Figure 17 depicts the corresponding permanent magnet equilibrium position. The magnetic flux lines were computed for the magnet in the coil’s equilibrium position. Figure 18 depicts that the induced voltage is a function of the negative time derivative, the calculated variation in permanent magnetic flux as a function of time concerning the equilibrium state. The computed flux accurately reproduced the sinusoidal curve according to the above mentioned geometry. Figure 19 depicts the magnetic flux density in Tesla measured in mm as a function of the permanent magnet. The computed flux reasonably well and accurately reproduces a sinusoidal curve. The resulting voltage amplitude of 17 V, although the average absolute voltage is 15.25 V, is the lowest input value for an established rectifier circuit, which should be sufficient for the proposed harvester for an embedded smartphone self-charging application.

### 4.3. RRAM Joint Model Optimization

**Figure 20:**
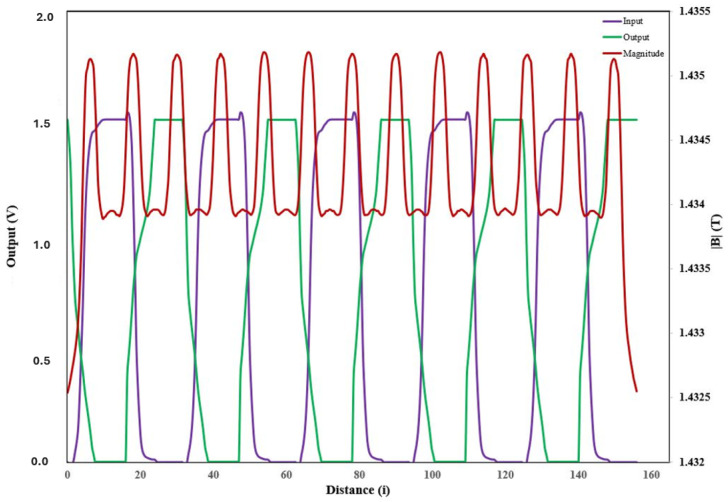
A computation of the in/out effectiveness of the proposed joint model amplification.

**Figure 21:**
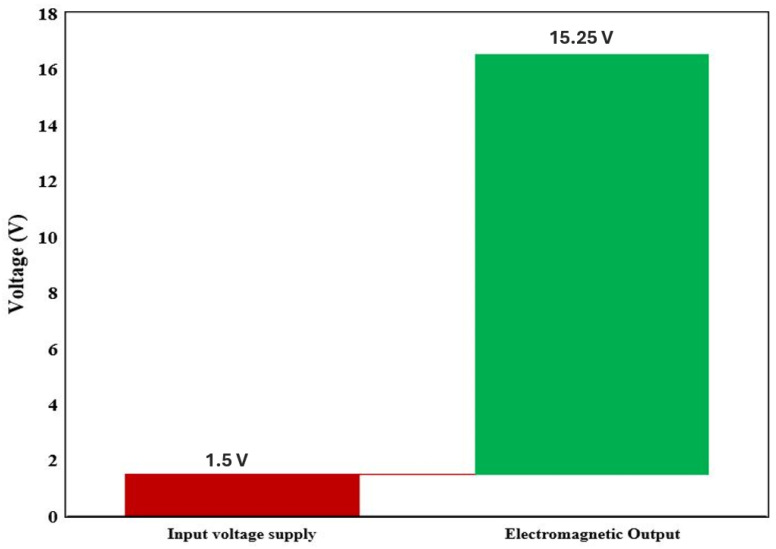
Measurement comparison: input vs. magnetism generated output.

In this part, we prove the efficacy of our suggested RRAM Joint Model Optimizations, which have been programmed to generate a self-charging process. The effectiveness of the proposed joint model in converting and amplifying the input energy is demonstrated in Figure 20, which graphically supports this significant voltage amplification. The suggested approach combines self-characterization processes to improve energy harvesting and storage efficiency. Figure 21 shows the difference between the 1.5 V input voltage and the 15.25 V magnetically produced output. Although the device uses a modest input supply of 1.5 V, magnetic induction generates an impressive 15.25 V output. This output exceeds the standard operating voltage of most embedded electronic devices, which typically operates within a 12V range. Table 6 shows the measured parameters of the proposed hybrid joint model. Based on these promising results, this evaluation indicates the effectiveness of the smartphone self-charging device. We believe that this performance validates our model as a more advanced and reliable alternative in the field of smart self-charging devices.

## 5. Conclusions

In this work, the possibility of harvesting human kinetic energy to power an embedded device has been simulated and evaluated. This simulation model is based on a developed energy generation system for integrated smartphone self-charging devices. The hybrid memristor-based self-charging system represents a promising approach for embedded smartphone energy solutions. It has a potential to drastically minimize the need for frequent charging and lead to a more energy-efficient and sustainable mobile technology ecosystem. The concept of using an RRAM device within an optimized cylinder-shaped permanent magnet in a CMOS memristor-based cell has been proposed. We employ a self-generation technique based on magnetic induction caused by the oscillation of a permanent magnet in a coil, and we propose a permanent magnet in the shape of a cylinder. This same approach was used in [19,20,22,23]. The design building block was simulated with the CMOS (130 nm) technology. To determine the induced voltage within the parameters of the finite-element method. Our research results show that we can use the significant thermoelectric voltage at the nanoscale to produce very intense transit-self-charging embedded devices. This approach, which incorporates energy harvesting and intelligent power management, can significantly prolong battery life and improve the sustainability of smartphone technology for self-charging device applications. We have also elaborated on how combining multiples of these blocks can form a self-charging device. The ultimate result might be an inventive transformation based on self-charging cell phones in the electronics sector. Finally, this breakthrough could revolutionize how smartphones manage energy, resulting in more autonomous and ecologically friendly devices. Future research will focus on further optimizing the architecture, addressing scalability difficulties, and investigating the integration of other developing energy sources for healthcare/serval different applications.

## Figures and Tables

**Table 1:** RRAM model specifications [39,43].

go = 5 × 10^−10^	Vo = 0.27 V	Io = 0.0003
vo = 0.8 m/s	B = 5.2	A = 2.1
gapini = 1.5 × 10^−10^	To = 300 K	Y= 22
gapmax = 1.5 × 10^−10^	tox = 6 nm	Io = 0.003
Ea = 0.6 eV	Rth = 1500 K/W	Y= Yreset = 15.3 nm

**Table 2:** Memristors with different voltage and dimension valves.

Supply Voltage	R1	R2
$V_{IN}$ = 0	$R_{ON}$	$R_{ON}$
$V_{IN}$ = 1	$R_{OFF}$	$R_{OFF}$
PMOS	W/L = 0.15 /0.13 μm	$V_{DD}$=1.5 V, $G_{ND}$ = 0 V

**Table 3:** Geometry specifics for the proposed magnet dimensions.

Specifications for Geometry	Length (mm)
L1	8 mm
L2	4 mm
L3	4 mm
L4	2 mm

**Table 4:** Simulated parameters of the suggested design.

Process File	Propsed 1T2R Design
Power supply	1.5 V, $G_{ND}$ = 0 V
Reference voltage	Vref = 0–1.5 V
Clock signal (CLK)	High = 1.5 V; low = 0 V
Power Consumption (µW)	∼17.75
Rise and fall time	5 ps

**Table 5:** Compared the proposed design to those proposed in the literature.

	This Work	2014 [23]	2023 [22]
Technology CMOS (nm)	130	90/180	130
Power supply	1.5 V	1.2/1.8 V	1.9 V
Power consumption Power Consumption (µW)	17.75	31/95	35.76
W/L	0.15 /0.13 µ	8/8	8
Vomax	≈1.5 V	≈1.2/1.8 V	≈1.9 V

**Table 6:** Hybrid joint model: 1T2R input supply vs. self-charged energy generation output.

Process Stage	Voltage (V)
1T2R Power supply	1.5 V, $G_{ND}$ = 0 V
Reference voltage	Vref = 0–1.5 V
Self-Charging energy generation via permanent magnet	∼Avg. 15.25 V
Voltage amplitude (permanent magnet)	17 V

## Data Availability

The original contributions presented in the study are included in the article, further inquiries can be directed to the corresponding author.
